# Locked-In Syndrome: A Rare Manifestation of Neuropsychiatric Lupus

**DOI:** 10.7759/cureus.62591

**Published:** 2024-06-18

**Authors:** Laura Polhemus, Divya Singh, Adam A Awad, Sandra Samuel, Navreet T Chennu, Gavin Defisser, Wilson Rodriguez, Jafar Kafaie

**Affiliations:** 1 Neurology, Saint Louis University School of Medicine, St. Louis, USA; 2 Neurology, Saint Louis University Hospital, St. Louis, USA

**Keywords:** locked-in syndrome, pontine edema, brainstem lesion, neuropsychiatric systemic lupus erythematosus (npsle), lupus cerebritis

## Abstract

Neuropsychiatric systemic lupus erythematosus (SLE) is a rare condition that has a multitude of mechanisms resulting in the emergence of variable clinical presentations. We describe a peculiar case of a 33-year-old female with a history of SLE presented with two weeks of fever, headache, and vomiting. On admission, she became obtunded and was emergently intubated. Initial lumbar puncture revealed pleocytosis (46% neutrophils, 320 corrected nucleated cells/μL), elevated protein (244 mg/dL; normal, 15-40 mg/dL), normal glucose (63 mg/dL), and negative cultures. Empiric acyclovir, ampicillin, ceftriaxone, and vancomycin were initiated without clinical improvement. Neurological examination was notable for limited ability to follow commands, vertical nystagmus, horizontal gaze palsy, diffuse hyperreflexia, and quadriparesis. Electroencephalogram (EEG) was consistent with diffuse encephalopathy. Brain magnetic resonance imaging demonstrated restricted diffusion and contrast enhancement in the posterior and central pons with edema. A cerebral angiogram showed no signs of vasculitis. Treatment with intravenous (IV) methylprednisolone 1 g and IV immunoglobulin 2 g/kg was initiated for five days. Despite these interventions, no discernible clinical improvement was observed, prompting the commencement of 500 mg/m^2^ cyclophosphamide and daily maintenance of IV methylprednisolone at 2 mg/kg. A repeat MRI three weeks later revealed a marked reduction in the size of the lesion involving the pons. The patient also improved clinically over the month with successful extubation, complete return in mental capabilities, and the ability to ambulate short distances with assistance.

## Introduction

Systemic lupus erythematosus (SLE) is a multi-system autoinflammatory condition that, when involved in the nervous system, can have varied neuropsychiatric manifestations, often referred to as neuropsychiatric SLE (NPSLE). It is estimated to occur in more than half of SLE individuals at some point in their lifetime, typically with a higher prevalence in females and people of African or Asian descent [1]. The American College of Rheumatology (ACR) nomenclature for NPSLE provides case definitions for 19 neuropsychiatric syndromes divided into central and peripheral nervous systems [2]. 

The most common NPSLE presentations involving the central nervous system are headaches followed by seizures and cerebrovascular diseases. Other common ones include aseptic meningitis, demyelinating conditions, movement disorders, encephalopathy, and anxiety [2]. Unique cases such as Cotard’s syndrome as a manifestation of lupus affecting the non-dominant frontotemporal and parietal lobes have also been reported [3]. Brainstem syndromes consisting of intractable hiccups, paraplegia, cranial neuropathies affecting the sixth nerve, internuclear ophthalmoplegia, and Sixteen syndrome have been described with involvement of medulla oblongata and pontobulbar region [4,5].

As there are no specific criteria or reliable biomarkers, the diagnosis of NPSLE can be challenging and is based on the principle of exclusion. Confounding illnesses, such as infection, electrolyte abnormalities, mass lesions, demyelinating conditions, and primary psychiatric disorders, are, thus, evaluated [6]. Serological studies, such as positive anti-dsDNA, anti-SS(A), anti-SS(B), anti-Smith, and anti-neuronal DNA, offer supportive evidence but are not diagnostic [7]. Brain parenchymal and vascular imaging are essential to localizing and deciphering etiologies of NPSLE [8]. Our case report highlights a unique but dreaded clinical presentation of partial locked-in syndrome secondary to NPSLE. We compare our differential diagnoses of pontine edema to prior studies describing brainstem pathology and abnormal brain imaging in the setting of lupus.

## Case presentation

**Figure 1:**
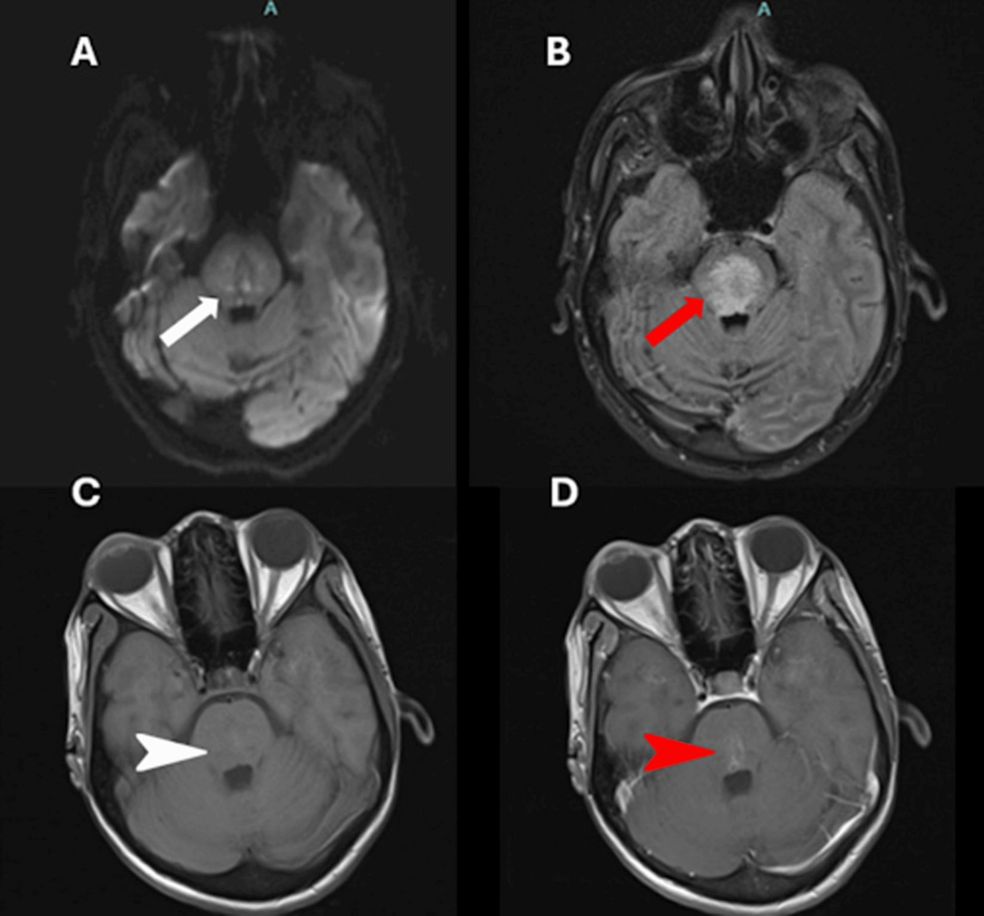
Initial brain MRI with and without contrast (A) The diffusion-weighted image sequence showing scattered diffusion restriction in the pons (white arrow). (B) Axial FLAIR demonstrating significant hyperintensity in pons (red arrow). (C,D) T1 sequence of pons without contrast (white arrowhead) and T1 post-contrast showing partial contrast enhancement in the pons (red arrowhead).

A 33-year-old female with an established diagnosis of SLE on hydroxychloroquine (HCQ) presented with a subacutely evolving fever, generalized weakness, headache, nausea, and vomiting over two weeks. On admission, she had a maximum temperature of 105°F (40.6°C), worsening mental status, and was emergently intubated for airway protection. Immediate lumbar puncture (LP) revealed neutrophilic pleocytosis (320 nucleated cells/μL, 46% segmented neutrophils), elevated protein (244 mg/dL; normal, 15-45 mg/dL), normal glucose (63 mg/dL), negative cultures, and meningitis-encephalitis polymerase chain reaction (PCR) panel. Empiric acyclovir, ampicillin, ceftriaxone, and vancomycin were initiated for suspected meningitis without clinical improvement over the following four days. Neurological examination was pertinent for limited ability to follow commands, vertical downbeat nystagmus, limited upward gaze with horizontal gaze palsy, hyperreflexia, and quadriparesis. Electroencephalogram (EEG) was consistent with diffuse encephalopathy. Brain magnetic resonance imaging (MRI) with and without contrast on the fourth day of hospitalization demonstrated restricted diffusion and contrast enhancement in the central and posterior pons with associated edema (Figure 1). This prompted a computed tomography (CT) angiography with similar findings and then a cerebral angiogram, which was unremarkable for signs of vasculitis or thrombosis.

Given the established diagnosis of SLE, treatment with intravenous (IV) methylprednisolone 1g daily and IV immunoglobulin (IVIG) 0.4 mg/kg daily was initiated for five days (for a total of 2 g/kg IVIG). Despite these interventions, no discernible clinical improvement was observed. Repeat LP after the aforementioned treatment was significant for lymphocytic pleocytosis (16 WBC, 86% lymphocytes), elevated protein (107 mg/dL), normal glucose (41 mg/dL), increased IgG (22.6 mg/dL; normal, 0.8-7.7 mg/dL), and negative meningitis-encephalitis PCR panel. IV cyclophosphamide (CYC) 700 mg (500 mg/m^2^) was given once. The patient continued IV methylprednisolone 2 mg/kg daily, tapered to 60 mg daily over three weeks, and included enteral HCQ 200 mg daily. During this time, further autoimmune workup revealed negative antiphospholipid antibodies, elevated ANA titer (1:2560; <1:40 negative), and positive ANA HEp-2 IgG antibodies. Cerebrospinal fluid (CSF) studies for autoimmune encephalitis and demyelinating diseases were unremarkable. 

**Figure 2:**
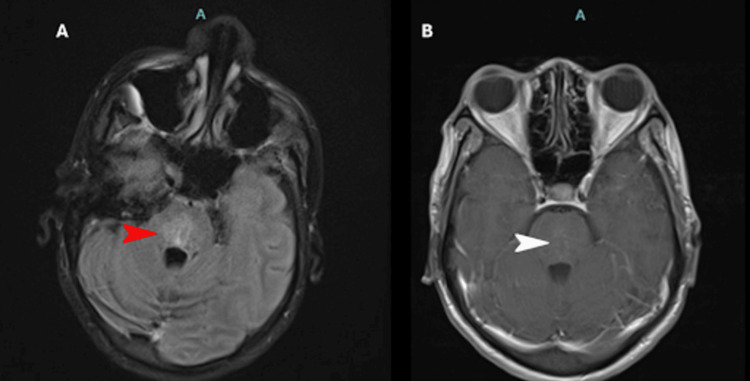
Brain MRI with and without contrast three weeks after admission (A) Decreased pons hyperintensity in axial FLAIR sequence (red arrowhead). (B) Minimal contrast enhancement compared with initial brain MRI (white arrowhead).

A repeat MRI of the brain three weeks following the above treatments (Figure 2) demonstrated interval improvement of edema and reduction of previously seen pontine enhancement. The neurological examination also improved gradually over this time, with successful extubation, complete return in mental capabilities, and the ability to ambulate short distances with assistance. The patient was given another treatment of IV CYC 770 mg (500 mg/m^2^ + 10%), four weeks after the first, before discharge to a long-term acute care facility. Her discharge medications included enteral prednisone, starting at 40 mg daily, tapered over six weeks to maintenance 10 mg daily, enteral HCQ 200 mg daily, and IV CYC 500 mg/m^2^ infusion every four weeks.

## Discussion

NPSLE has a broad spectrum of clinical presentations but is rarely cited for causing localizing brainstem symptomatology. Our patient exhibited unique MRI findings of diffusion restriction and partial contrast enhancement in the pons, presenting phenotypically as partial locked-in syndrome. Differentials for the radiographic findings were acute ischemia, sequelae of vasculitis, demyelinating disease, central pontine myelinolysis (CPM), posterior reversible encephalopathy syndrome (PRES), and CLIPPERS syndrome. We aim to compare our patient’s clinical course and radiographic findings to other reported cases of NPSLE and attempt to justify the etiology of our patient’s lesion. 

Only some reports regarding MRI patterns in NPSLE have been published. Structural involvement of NPSLE is commonly demonstrated as diffuse cortical atrophy, ventricular dilatation, and white matter disease seen as T2 FLAIR hyperintensities [9]. Early studies have shown a higher correlation of signal intensity in the subcortical white matter, frontal cortex, pons, and basal ganglia in patients with NSPLE than non-NP SLE [10]. One study showed that cerebral blood flow is significantly decreased in the thalamus, right anterior insula, and notably the pons in patients with NPSLE and SLE compared to healthy controls, indicating possible blood-brain barrier (BBB) disruption in these regions [11]. A larger retrospective cross-sectional study performed years later found that MRI abnormalities were present in 59.3% of NPSLE patients within six months of symptom onset. They found that white matter lesions correlated most with cognitive dysfunction, large vessel disease and microbleeds with cerebrovascular syndromes, and inflammatory-like lesions with myelopathy [12]. A cohort study demonstrated that 25% of patients with NPSLE had cerebral atrophy (18%) or focal white matter lesions (8%); however, localization to the brainstem is not defined [13].

Ischemia related to prothrombotic states has been more extensively studied in patients with NPSLE, as up to 15% of cases present as cerebrovascular accidents [2]. A series of 118 NPSLE patients studied MRI findings and found that approximately 66% of patients had ischemic changes on MRI, including pontine regions, which correlated with antiphospholipid (aPL) antibodies and C3 [14]. A large case-control study of 256 SLE patients showed that abnormal MRI findings, including large territorial, lacunar, localized cortical, border zone infarcts, basal ganglia lesions, and stenotic arterial lesions, are more common in SLE patients with antiphospholipid syndrome than without [15]. One case report demonstrated a meningitic presentation similar to our patients’ with corresponding punctate restricted diffusion in the supra and infratentorial parenchyma. However, no lesions in the pons or corresponding focal neurological deficits were identified [16]. Our patient had negative aPL antibodies, and imaging was not consistent with a definite vascular territory infarct, and radiographic improvement within three weeks made thromboembolic stroke a less likely cause. 

Cerebral vasculitis and vasculopathy, also tightly related to ischemic lesions, were further differentials explored in our patient. Cerebral vasculitis is associated with T2 hyperintensities on MRI, with 30-35% involving typically the white matter tracts [9]. Endothelial cell antibodies (AECA) and aPL antibodies cause cell wall damage and the production of interleukins and cytokines that further potentiate BBB dysfunction, thus causing diffusion restriction on MRI [17]. Formal cerebral angiography eventually ruled out vasculitis as a potential source of pontine edema in our patient. 

Demyelinating lesions typically produce T2 hyperintensities with contrast enhancement on T1 within the supratentorial periependymal regions, optic tracts, and spinal cord and often have correlating myelopathy and positive antibody studies [9,18]. Our patient’s serum testing for neuromyelitis optica (NMO) and myelin oligodendrocyte (MOG) antibodies were negative, and suspicion for seronegative disease was lower on the differential. CPM has not been correlated with NPSLE to date, and our patient had no rapid correction of sodium or history of chronic liver disease to suggest it. Interestingly, SLE is the most prevalent autoimmune condition associated with PRES, and some authors argue that PRES should be added to its list of neuropsychiatric manifestations [19]. In our case, we would have expected to see electrographic or clinical seizures and our patient’s edema to resolve more rapidly.

Our patient’s syndrome and imaging findings had subacute progressive improvement after treatment with immunomodulating drugs. Ultimately, the etiology of our patient’s lesion is still up for debate. However, it seems that immunosuppression was a catalyst in reducing her cerebral edema. Although serological testing yielded negative results for specific proinflammatory markers of SLE related to arterial damage (and thus BBB damage), notably aPL antibodies, the data remains scarce regarding other immune proteins that may have been present and the culprit for cerebral edema.

## Conclusions

Neuropsychiatric lupus has varied phenotypic manifestations, with few reported cases of brainstem pathology. Our case is an extremely unique clinical presentation of partial locked-in syndrome secondary to NPSLE, emphasizing the diversity and severity of this disease. While the investigations and aggressive treatment undertaken with high-dose steroids and CYC are similar to the many lupus flare cases, the diagnostic challenge remains the crux of the disease. Furthermore, the pathophysiology behind the pons being at risk is intriguing and offers an area for further research.
